# Supplementary material for: Sample Preparation Strategies for Antibody-Free Quantitative Analysis of High Mobility Group Box 1 Protein
Source: Pharmaceuticals (Basel). 2021 Jun 3;14(6):537. doi: 10.3390/ph14060537 (PMC8230321; doi:10.3390/ph14060537)
Supplement: Supplementary file 1 [file pharmaceuticals-14-00537-s001.zip › pharmaceuticals-1248408-supplementary.pdf]

Supplementary Methods

Immunoprecipitation with Anti-HMGB1 and Anti-Haptoglobin Antibodies

Dynabeads ® M-280 tosylactivated (Invitrogen, Carlsbad, CA, USA), 5 mg, was coupled to 50 µg anti-HMGB1 antibodies or 100 µg anti-Haptoglobin antibodies (prepared as described in Materials and Methods) overnight, according to the manufacturer's protocol. The resulting bead concentration was 20 mg/mL. For the anti-HMGB1 antibody assay, 50 µL of the coated bead solution was added to 250 µL of the sample, and incubated with rotation for 1 h at RT. Subsequently, the beads were washed once with 200 µL phosphate-buffered saline (PBS) w/0.05% Tween, once with 200 µL PBS, once with 10 mM Tris-HCl (pH 7.4), and finally with 200 µL ammonium bicarbonate (AMBIC) 100 mM following Levernæs et al. [1], with minor modifications. The beads were then resuspended in 20 µL of 100 mM AMBIC before on-bead trypsin digestion as described above, but with iodoacetamide (IAA) as the alkylation reagent.

For the anti-haptoglobin assay, samples were prepared by adding recombinant dsHMGB1 to plasma, followed by incubation for 30 min at 37 °C, as described by Yang et al. [2]. The samples were diluted 1:40 with PBS, and 50 µL of the coated bead solution was then added to 200 µL of the diluted sample, and incubated with mixing at 37 °C. The beads were then washed three times with 1 mL Buffer E from Invitrogen, before eluting the captured proteins by adding 50 µL of 200 mM glycine (pH 2.6), vortexing, and 5 min incubation. This was repeated three times, yielding a total of 150 µL eluate. Tris-HCl (50 µL, 1 M, pH 7.5) was added to neutralize the sample.

Precipitation with Perchloric Acid (PCA), Followed by Filter-Aided Sample Preparation (FASP)

PCA (13.7% w/w) was prepared as described by Gaillard et al. [3], as follows: 1.26 vol of 70% PCA was added to 8.96 vol of water. Then, 185 µL plasma, spiked with 1 µg recombinant HMGB1, was added to 15 µL of 5 M NaCl and 100 µL of 13.7% PCA to obtain a final concentration of 5% PCA. The sample was vortexed for 10 s, incubated on ice for 10 min, and centrifuged for 10 min at 15000 ×*g* at 4 °C. The supernatant was transferred to a 5 kDa MW cut-off filter (Ultrafree®-MC, Millipore, Bedford, MA, USA). The filter was assembled with a sample tube, and centrifuged for 40 min at 5000 ×*g* at RT. Addition of 200 µL AMBIC for washing, followed by 40 min of spinning at 5000 ×*g* at RT, was repeated until pH > 7 was attained.

Subsequently, FASP was performed as follows: proteins retained on the filter were reduced by adding 30 µL of 30 mM DL-dithiothreitol (DTT), and 30 min incubation at RT, followed by the addition of 30 µL of 90 nM N-ethylmalemide (NEM) for 10 min at RT. The proteins were digested with 30 µL of 0.25 mg/mL trypsin with 20 mM $CaCl_2$ overnight at 37 °C. The filter was then transferred to a clean sample tube, and centrifuged for 2 min at 1000 ×*g*. Trifluoroacetoc acid (TFA) (1 µL) and acetonitrile (ACN) (5 µL) were subsequently added to the sample.

Digestion of Proteins with Acetic Acid

The method for digestion using acetic acid is described in a patent [4]. Recombinant HMGB1 (10 µL, 200 µg/mL), neat or spiked, was analyzed in a total volume of 200 µL plasma, at a concentration of 10 µg/mL. DTT (10 µL, 200 mM) was added for 10 min, followed by 10 µL of 120 mM NEM for 10 min. Subsequently, 33 µL or 66 µL of 100% acetic acid, and 157 µL or 154 µL water were added, respectively, resulting in 15% acetic acid in the sample. Incubation for 2 h at 110 °C digested the proteins before separation by high performance liquid chromatography (HPLC).

Separation of Acid-Digested Proteins with HPLC

The digested samples were injected into the HPLC system, equipped with a UV detector, and fraction collector described earlier. The sample (100 µL) was separated with an Inertsil ODS-4 C18 column, 21 × 150 mm (GL Sciences, Eindhoven, The Netherlands) at 45 °C. A gradient was run with solvent A (0.1% TFA) and solvent B (ACN) at a flow rate of 0.2 mL/min, as follows: 1–60% ACN for 1–40 min, 60–70% for 40–45 min, and 70% ACN for 45–50 min. The UV detector was set at 280 nm. Several fractions were collected in conical 1.5 mL PP-tubes, initially between 3.75–33 min, and in later experiments, every two minutes between 17.8–25.8 min.

The fractions were dried using a miVac concentrator with Speed Trap (Genevac Ltd., Ipswich, UK) at 80 °C for 1 h. For analysis of acid-digested peptides, the dried peptides were resuspended in FA and analyzed using HRMS. Otherwise, the peptides were resuspended in 130 µL of 80% Tris-HCl and 20% ACN, and digested with 20 µL of 0.5 mg/mL trypsin with 200 mM $CaCl_2$ at 37 ℃ for 1 h. The samples were evaporated to dryness at 80 ℃ for 30 min, and resolved in 25 µL of 1% FA before HRMS analysis.

References

1. Levernæs, M.C.S.; Broughton, M.N.; Reubsaet, L.; Halvorsen, T.G. To elute or not to elute in immunocapture bottom-up LC-MS. *J Chromatogr B Analyt Technol Biomed Life Sci* **2017**, *1055-1056*, 51-60, doi:10.1016/j.jchromb.2017.03.044.
2. Yang, H.; Wang, H.; Levine, Y.A.; Gunasekaran, M.K.; Wang, Y.; Addorisio, M.; Zhu, S.; Li, W.; Li, J.; de Kleijn, D.P.V.; Olofsson, P.S.; Warren, H.S.; He, M.; Al-Abed, Y.; Roth, J.; Antoine, D.J.; Chavan, S.S.; Andersson, U.; Tracey, K.J. Identification of CD163 as an antiinflammatory receptor for HMGB1-haptoglobin complexes. *JCI Insight* **2016**, *1*, doi:10.1172/jci.insight.85375.
3. Gaillard, C.; Borde, C.; Gozlan, J.; Maréchal, V.; Strauss, F. A high-sensitivity method for detection and measurement of HMGB1 protein concentration by high-affinity binding to DNA hemicatenanes. *PLoS One* **2008**, *3*, e2855, doi:10.1371/journal.pone.0002855.
4. Kwon, J.; Lee, T. Protein cleavage at aspartic acid using chemical reagents. WO2006031063A1, **2006**.

(a)

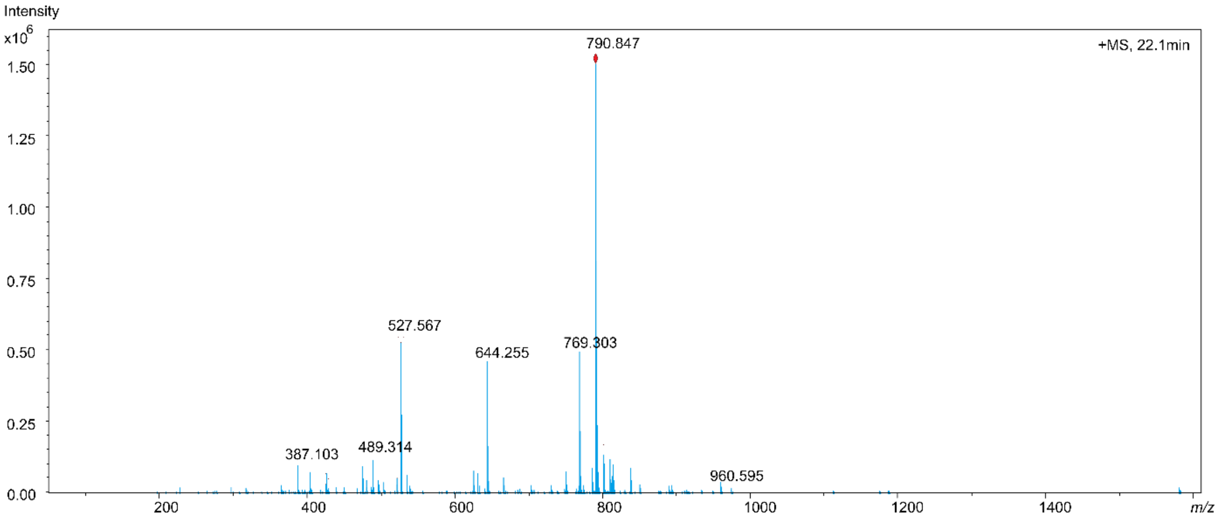

(b)

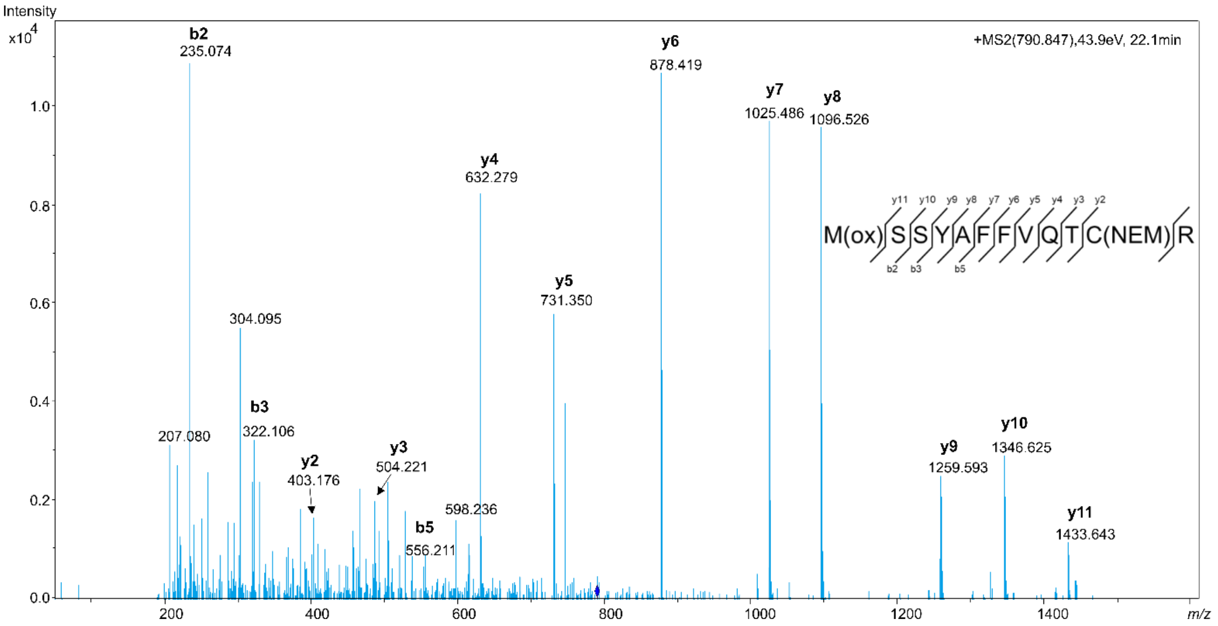

Supplementary Figure S1

Mass spectrum from high resolution mass spectrometry (HRMS), identifying high mobility group box 1 (HMGB1) peptide MSSYAFFVQTCR alkylated with N-ethylmaleimide (NEM) and with oxidized methionine (M(ox)) (a) Molecular ion mass spectrum showing the peptide mass $[M+2H]^{2+}$ with *m/z* 790.847, at 22.1 min. (b) Fragment ion mass spectrum of *m/z* 790.847 showing series of y- and b-ions identifying the amino acid sequence.

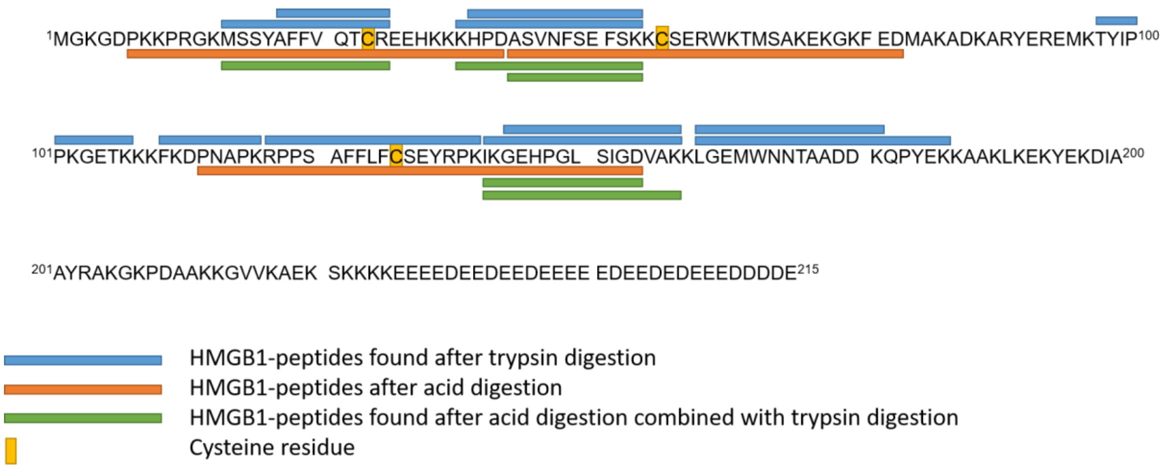

Supplementary Figure S2

The amino acid sequence for high mobility group box 1 (HMGB1) protein with the peptides found using different digestion methods.

(**a**)

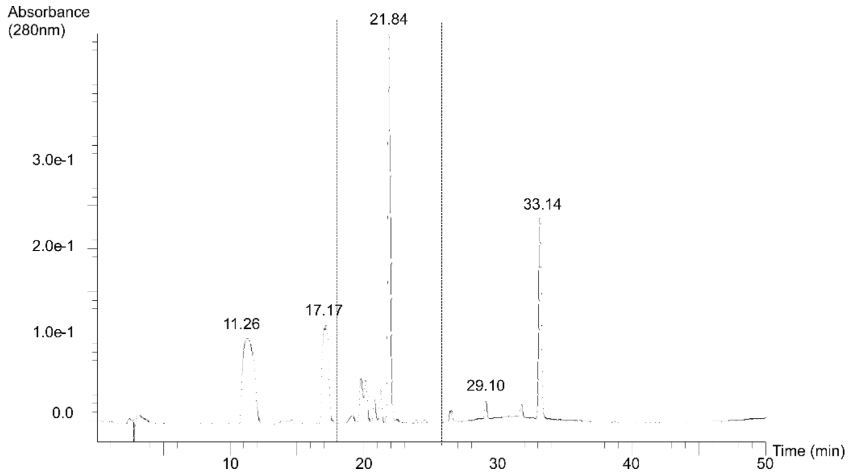

(**b**)

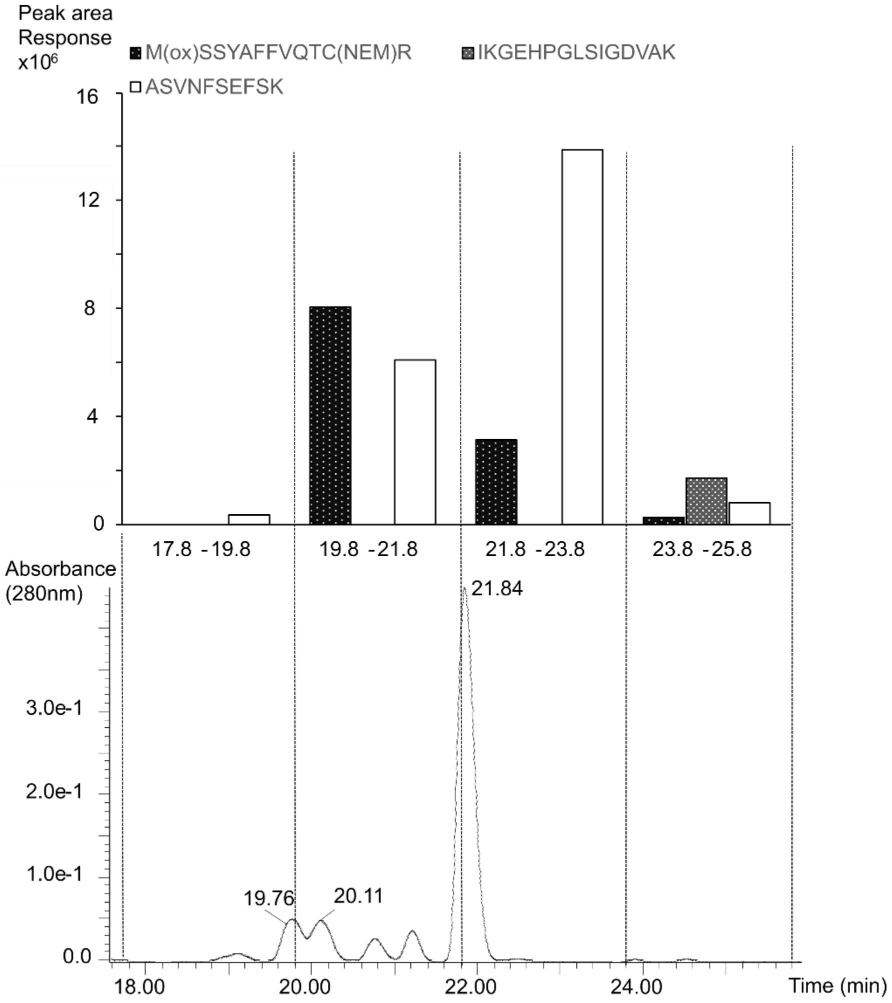

Supplementary Figure S3

High performance liquid chromatography (HPLC) separation of acid digested high mobility group box 1 (HMGB1). (**a**) Chromatogram from HPLC. Peptides from HMGB1 were mainly found in the fraction eluted between 17.8 min and 25.8 min. (**b**) Peak area response for four different HMGB1 peptides in the 2-min-fractions between 17.8 min and 25.8 min.
